# Mulberry ERF Transcription Factor MnERF23 Is a Positive Regulator of Plant Drought Tolerance

**DOI:** 10.3390/plants15182868

**Published:** 2026-09-19

**Authors:** Xueqiang Su, Rong Zhou, Manli Zhao, Cuimin Xu, Qianqian Lu, Ran Zhang, Taichu Wang, Ruixue Li

**Affiliations:** Sericultural Research Institute, Anhui Academy of Agricultural Sciences, Hefei 230000, China

**Keywords:** mulberry, ERF genes, drought stress, transcriptional regulation

## Abstract

Mulberry (*Morus alba* L.) is an important economic tree species, and its leaves are increasingly being used to prepare healthy dishes in addition to serving as food for silkworms. Drought is one of the main stress factors affecting the quality of mulberry leaves; however, the molecular mechanisms through which mulberry plants respond to drought stress are currently unclear. The AP2/ERF gene family is involved in plant growth, development and stress response processes. In response to drought stress, the expression of mulberry *MnERF23* is induced. The overexpression of *MnERF23* in *Arabidopsis thaliana* enhances drought tolerance, increases root length, and activates the expression of stress-related genes in transgenic plants. The transient overexpression of *MnERF23* in mulberry leaves can reduce stress-related damage in transgenic seedlings and promote better growth. In this study, RNA-seq analysis of mulberry leaves transiently overexpressing *MnERF23* in both normal and drought environments revealed 470 and 687 DEGs, respectively. In the DS-1305 vs. DS-ERF23 comparison, genes related to ROS clearance, cell wall precursors, and lignin synthesis were up-regulated. Further analysis revealed that *Mn4CL3* is an up-regulated differentially expressed gene and that the promoter retains a DRE/CRT *cis*-acting element. The Y1H and dual-luciferase assay results reveal that *MnERF23* can positively regulate *Mn4CL3* in combination with DRE/CRT, synergistically enhancing the drought tolerance of mulberry. We elucidated the regulatory mechanism through which *MnERF23*-dependent DRE/CRT *cis*-acting elements promote *Mn4CL3* expression and enhance drought tolerance in mulberry. This study contributes to the theoretical research on the regulatory network of plant abiotic stress, providing a theoretical basis and technical support for forest genetic breeding.

## 1. Introduction

The global trend of farmland drought is becoming increasingly severe, with approximately 50% of farmland in a dry or semiarid state [1,2]. In plants, drought mainly causes water deficiency and changes the osmotic potential of cells, leading to damage to the photosynthetic system, inhibition of secondary metabolism and reduced yield; severe cases can cause death [3]. The main agricultural product of the mulberry plant is leaves, which are relatively susceptible to drought stress which can result in reduced yields [4]. Therefore, to cope with future climate change and ensure increased agricultural production, improving the ability of crops to resist abiotic stress, especially drought stress, has been given high priority.

The responses of plants to drought stress can be divided into five main steps: Signal perception, signal transduction, transcriptional regulation, expression of drought-responsive genes, and activation of physiological responses. Transcription factors play important roles in this process [5]. Researchers have identified members of multiple transcription factor families across different species involved in the drought stress response. In tomato, *SlMYB1L* inhibits the expression of the abscisic acid (ABA) catabolic gene *SlCYP707A3*, regulates endogenous ABA levels, and enhances plant drought tolerance [6]. *OsbZIP27* enhances drought tolerance in rice by activating the expression of genes such as *OsHAK1* and *OsGLN* [7]. Overexpression of *HvHSFA2e* in transgenic barley significantly increases the chlorophyll content and membrane stability and reduces lipid peroxidation and ROS accumulation [8]. In *Arabidopsis thaliana*, *AtWRKY43* enhances plant drought tolerance by positively regulating *AtSWEET5* expression [9]. Our previous research revealed that mulberry *MaWRKYIIc7* can activate the expression of *MaN*CE*D1* and *MaRD29A*, synergistically improving the drought tolerance of mulberry [10]. In addition, the *AP2/ERF* gene family plays important roles in regulating plant secondary metabolism, growth and development, and the response to abiotic stress [11].

In accordance with the number of structural domains and phylogenetic relationships, *AP2/ERF* is divided into four subfamilies: *AP2*, *RAV*, *DREB*, and *ERF* [12]. Numerous studies have reported that *ERFs* are involved in the responses to various abiotic stresses, including drought stress [13,14]. *TaERFL1a* can up-regulate the expression of genes such as *GPX*, *DHAR* and *MDHAR* and activate the ascorbic acid and glutathione biosynthesis pathways [15]. *OsERF71* is induced by ABA and drought stress and can regulate the expression of ABA and proline biosynthesis genes, promoting tolerance to drought stress in rice [16]. *ERFs* can also mediate the plant drought stress response by regulating lignin biosynthesis, much like *PtoERF15* affects the development of xylem vessels to maintain stem water potential and ultimately improve the drought tolerance of poplar [17]. In summary, *ERFs* are involved in the plant drought stress response and have enormous potential for drought tolerance.

The whole-genome identification of the AP2/ERF gene family in mulberry has been completed, and the biological functions of some genes, such as *MaERF-B2-1* and *MaERF-B2-2*, have been elucidated [18,19,20]. Given the gaps in research on *ERF* transcription factors that mediate tolerance to drought stress in mulberry, we identified an *ERF* gene (*MnERF23*) that may be involved in regulating the drought stress response and investigated its function. *MnERF23* can increase the drought tolerance of transgenic *Arabidopsis thaliana* and transiently overexpressing mulberry, inhibit the accumulation of MDA in transgenic plants, and increase the activity of ROS scavenging-related enzymes. Further research revealed that *MnERF23* was responsible for activating *Mn4CL3* expression under drought stress, which is involved in drought tolerance in mulberry. Our research contributes to the understanding of *ERF*-mediated drought stress response regulatory network in mulberry, providing a theoretical basis for enhancing plant drought tolerance through lignin accumulation.

## 2. Results

### 2.1. MnERF23 Is Potentially Involved in Regulating the Drought Stress Response

**Figure 1:**
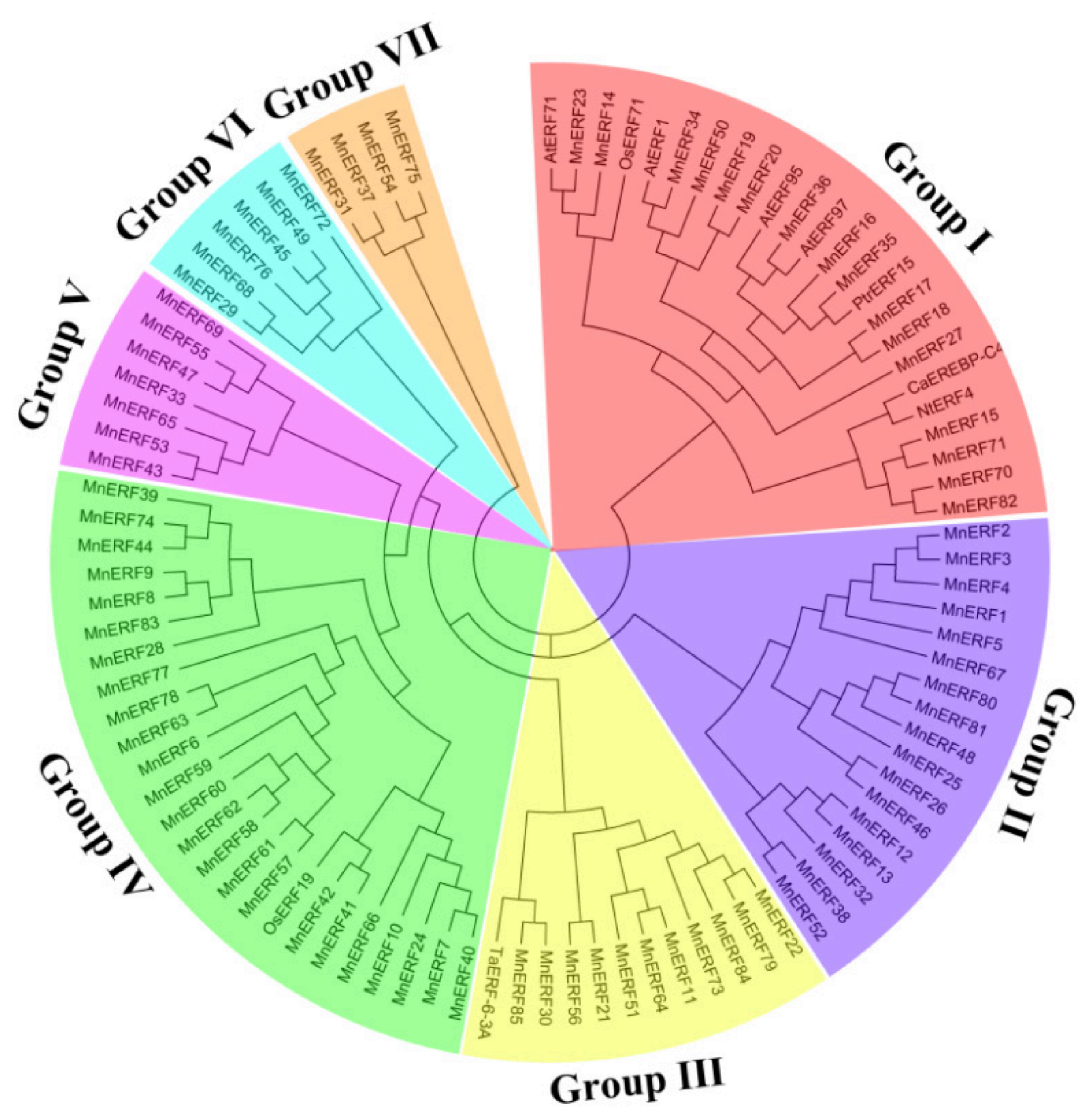
Phylogenetic tree of the relationships among 85 *MnERFs* in mulberry and *ERFs* with known functions in *Arabidopsis thaliana*, *Oryza sativa*, *Populus trichocarpa*, *Triticum aestivum*, *Nicotiana tabacum* and *Capsicum annuum*.

**Figure 2:**
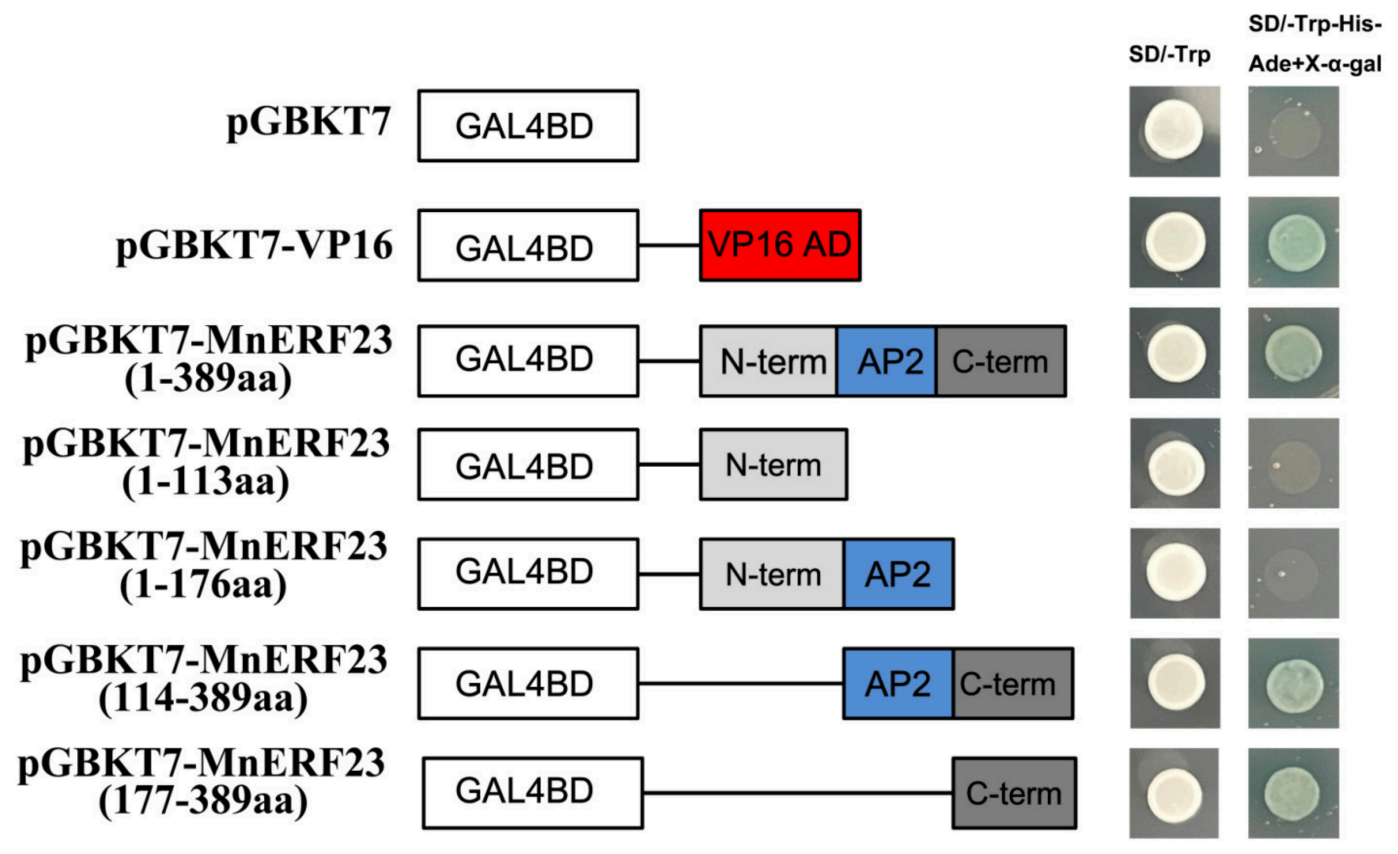
Transcriptional activation activity of MnERF23. GAL4 DNA-BD was fused with MnERF23 (1-389 aa, 1-113 aa, 1-176 aa, 114-389 aa, 177-389 aa) and cultured on SD/-Trp and SD/-Trp/-His/-Ade+X-α-gal media at 30 °C. pGBKT7-VP16 was used as the positive control, and pGBKT7 was used as the negative control.

A phylogenetic tree of 85 *MnERF* and *ERF* genes with known functions in species such as *Arabidopsis thaliana*, *Oryza sativa* and *Populus trichocarpa* was constructed, and we identified 7 groups, namely groups I-VII (Figure 1). The results revealed that in group I, *MnERF14*, *23*, *34*, and *35* were clustered together with some functional genes related to osmotic stress (*AtERF71*, *OsERF71*, *PtrERF15*). *MnERF30* and *85* were in group III and were closely related to *TaERF-6-3A*. We analyzed the changes in the expression levels of *MnERFs* under drought stress using published transcriptome data (PRJNA692033) (Table S1) [21]. After 9 d of exposure to drought stress, 23 genes were up-regulated, and 26 genes were down-regulated. Among these genes, *MnERF23* and *MnERF34* were up-regulated, whereas the expression levels of *MnERF14* and *MnERF35* did not change upon exposure to stress (with low expression levels of *MnERF34* and *MnERF35* in leaves). Further analysis of the expression patterns of *MnERF14* and *MnERF 23* revealed that *MnERF23* expression was induced by drought stress and that its expression level increased with increasing duration of drought stress, peaking at 8 d (Figure S1). Changes in the expression pattern of *MnERF14* seemed to be weakly correlated with sustained drought stress. The above results revealed that *MnERF23* is a gene closely related to genes known to be involved in regulating the drought stress response. Sequence comparison revealed that *MnERF23* is a typical *ERF* gene, with alanine and aspartic acid at positions 14 and 19 of the AP2 domain, respectively (Figure S2). We then cultured yeast cells carrying pGBKT7-*MnERF23* (1-389 aa, 1-113 aa, 1-176 aa, 114-389 aa, 177-389 aa) recombinant plasmids on SD/-Trp/-His/-Ade+X-α-gal medium to investigate the transcriptional activity of *MnERF23* in the absence of bait protein (pGBKT7-VP16 was the positive control, and pGBKT7 was the negative control) (Figure 2). Yeast cells transformed with pGBKT7-*MnERF23* (1-389, 114-389, 177-389 aa) grew normally on the culture medium.

### 2.2. Overexpression of MnERF23 in Arabidopsis Thaliana and Mulberry Leaves Enhances Drought Tolerance

**Figure 3:**
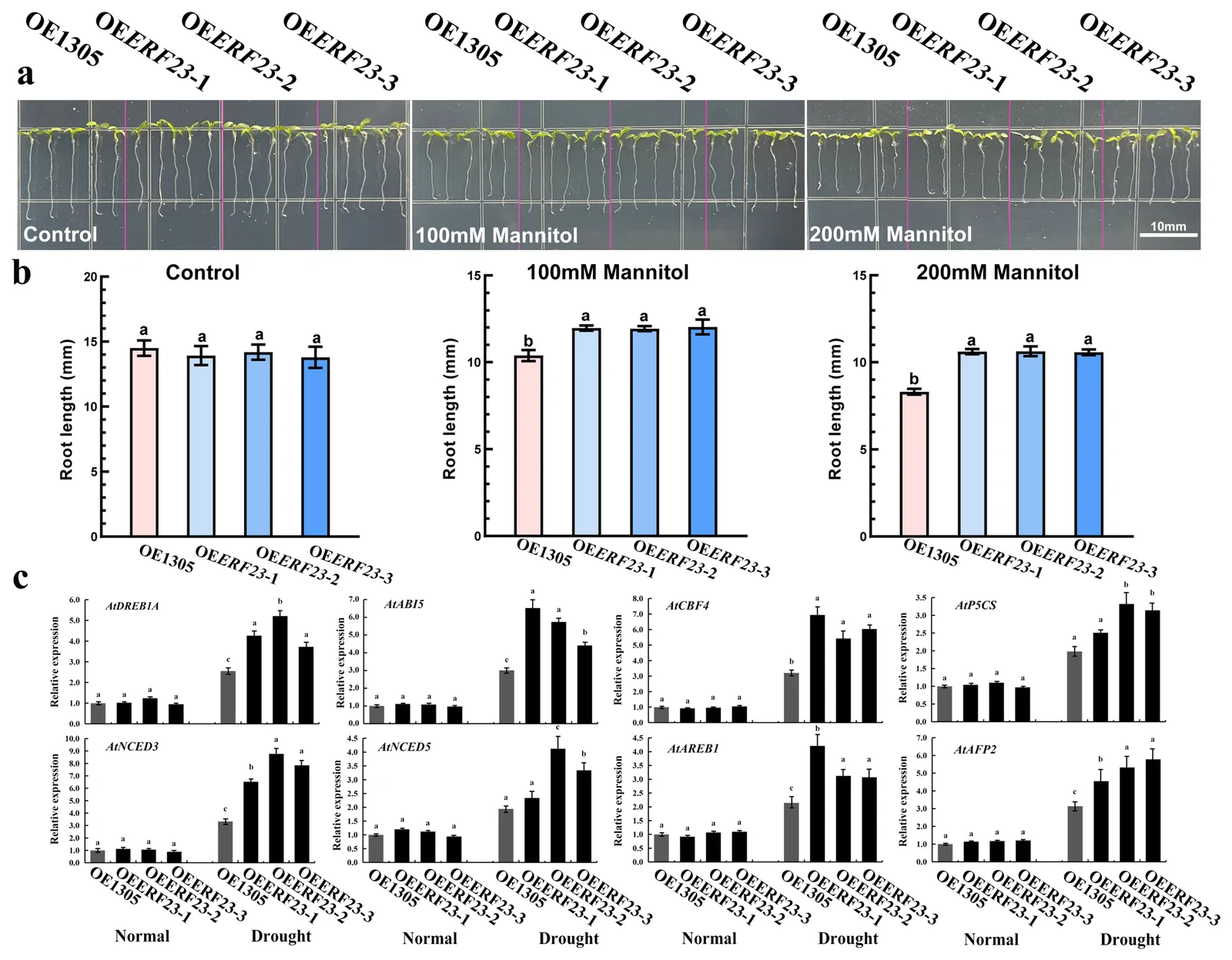
Overexpression of *MnERF23* in *Arabidopsis thaliana* enhances drought tolerance. (**a**) Effect of mannitol treatment on the growth of *MnERF23*-overexpressing transgenic *Arabidopsis thaliana* plants. (**b**) Determination of main root length after 5 d of mannitol treatment (0, 100, 200 mM). Different lowercase letters indicate significant differences between samples (*p* < 0.05), whereas the same letters indicate no significant differences. (**c**) Detection of the expression levels of drought stress-related genes in transgenic *Arabidopsis thaliana*. Different lowercase letters indicate significant differences between samples (*p* < 0.05), whereas the same letters indicate no significant differences.

*MnERF23* was overexpressed in wild-type *Arabidopsis thaliana*, and the plants in the control group were transformed with the pCAMBIA1305 empty vector, OE1305. Three transgenic lines with high expression levels of *MnERF23* were selected and named OE*ERF23*-1, 2, -3 (Figure S3). Mannitol is convenient for controlling concentration, and we use mannitol to simulate drought stress. When mannitol was not added to the culture medium, the root growth of OE1305 and OE*ERF23*-1, -2, -3 did not significantly change (Figure 3a). When the concentration of mannitol was 100 mM, the lengths of the roots of OE1305 and OE*ERF23*-1, 2, -3 decreased, but the lengths of the roots of OE*ERF23*-1, 2, -3 were greater than that of OE1305. Under 200 mM mannitol treatment, root elongation was inhibited in all lines, but the root lengths of OE*ERF23*-1, -2, -3 were significantly greater than that of OE1305 (Figure 3b). Further analysis revealed that the expression levels of genes related to osmotic stress, such as *AtDREB1A*, *AtABI5*, *AtNCED3*, were significantly increased in transgenic *Arabidopsis thaliana* (Figure 3c).

**Figure 4:**
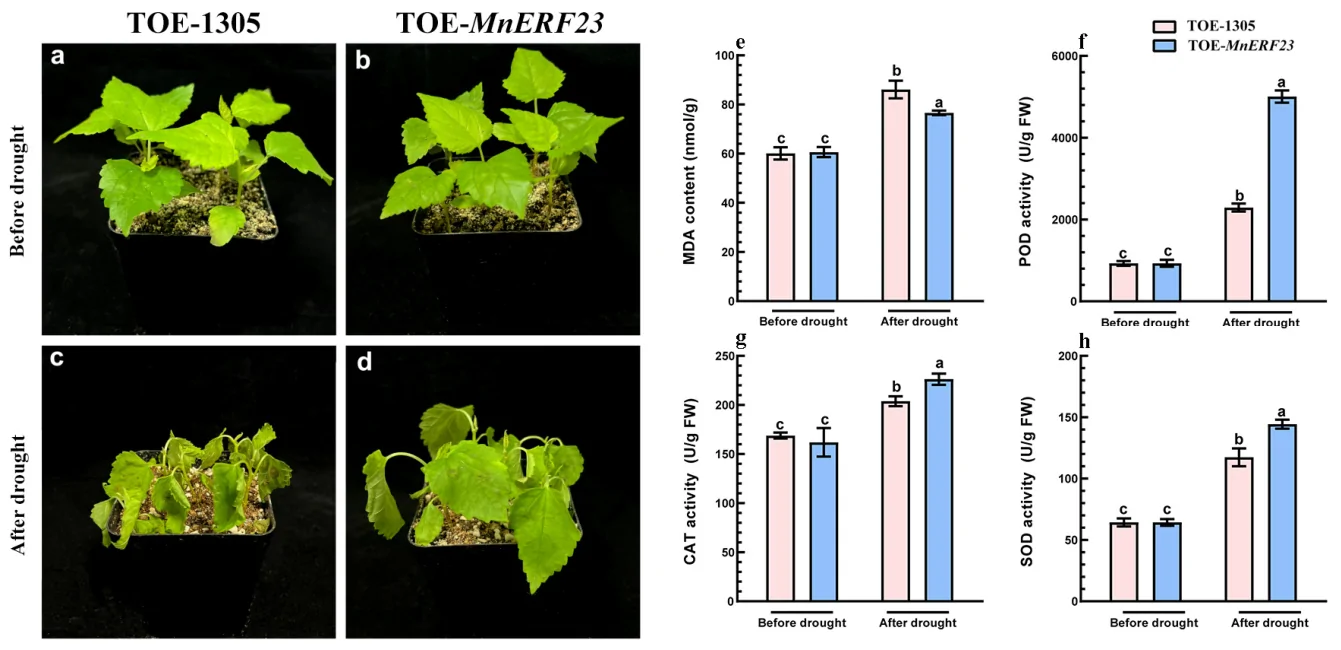
Phenotypic analysis of *MnERF23* transient overexpression in mulberry seedlings under drought stress and detection of stress-related physiological indicators. (**a**,**b**) Normally growing TOE-1305 and TOE-*MnERF23*. (**c**,**d**) TOE-1305 and TOE-*MnERF23* after drought stress treatment. (**e**) MDA content detection. (**f**) POD activity detection. (**g**) CAT activity detection. (**h**) SOD activity detection. Different lowercase letters indicate significant differences between samples (*p* < 0.05), whereas the same letters indicate no significant differences.

To clarify the function of *MnERF23*, we transiently overexpressed *MnERF23* in leaves of mulberry seedlings and labeled this group of leaves as TOE-*MnERF23* (the control group was generated using the pCAMBIA1305 empty vector, TOE-1305). Compared with TOE-1305, the transcript level of *MnERF23* in TOE-*MnERF23* remained higher at 10 d of drought stress (Figure S4). Both TOE-1305 and TOE-*MnERF23* presented significant stress phenotypes, with inhibition of normal plant growth, wilting of leaves and damage to upright stems. However, compared with TOE-1305, TOE-*MnERF23* exhibited milder stress symptoms, retained relatively higher water content and maintained its leaf status to some extent (Figure 4a–d and Figure S5). We examined the POD, SOD, and CAT activities of TOE-1305 and TOE-*MnERF23* and noted that the change patterns in the activity of these peroxidases were consistent (Figure 4f–h). Under drought stress, the activities of POD, SOD, and CAT in TOE-*MnERF23* were activated, and the activity in TOE-*MnERF23* was significantly greater than that in TOE-1305. MDA can reflect the degrees of lipid peroxidation and oxidative damage in organisms, which significantly increase under drought stress. The degree of oxidative damage in TOE-*MnERF23* was relatively low, and the content of MDA was lower than that in TOE-1305 (Figure 4e). These results indicate that the overexpression of *MnERF23* enhances the responses of *Arabidopsis thaliana* and mulberry seedlings to drought stress and reduces the adverse effects caused by stress.

### 2.3. Overview of RNA-Seq Results from Mulberry Leaves with Transient Overexpression of MnERF23

Our research reveals that *MnERF23* may be a positive factor regulating the drought stress response of mulberry. To elucidate the regulatory network and related molecular mechanisms involved in *MnERF23*, RNA-Seq analysis was conducted on TOE-*MnERF23* and TOE-1305 (PRJNA1404858). We established two comparison groups, CK-1305 vs. CK-ERF23 and DS-1305 vs. DS-ERF23 (each sample was set up with three biological replicates). Total RNA was extracted from 4 samples separately, after which a cDNA transcriptome sequencing library was constructed. After removing the adaptors and low-quality sequences, we obtained the total reads of each sample and mapped them onto the mulberry tree reference genome, and the percentages of mapped reads for the libraries ranged from 66.74 to 73.69%. We found that the GC contents of the 12 libraries ranged from 45.11 to 46.09%, and the Q20 and Q30 values were relatively similar between the libraries (Table S2). Among the total mapped reads, 92.63–93.26% of the reads were mapped to exonic regions, which constitutes the greatest proportion of the structure. The intergenic region had the smallest percentage, with only approximately 2.55–2.9% of the reads being mapped to this region (Figure S6). The 4 libraries were further divided into two comparison groups: CK-1305 vs. CK-ERF23 and DS-1305 vs. DS-ERF23. We identified 470 DEGs (226 up-regulated and 244 down-regulated) from CK-1305 vs. CK-ERF23, while DS-1305 vs. DS-ERF23 had 554 up-regulated and 133 down-regulated expressed genes (Figure S7).

### 2.4. DEG Functional Enrichment and Related Metabolic Pathway Analyses

**Figure 5:**
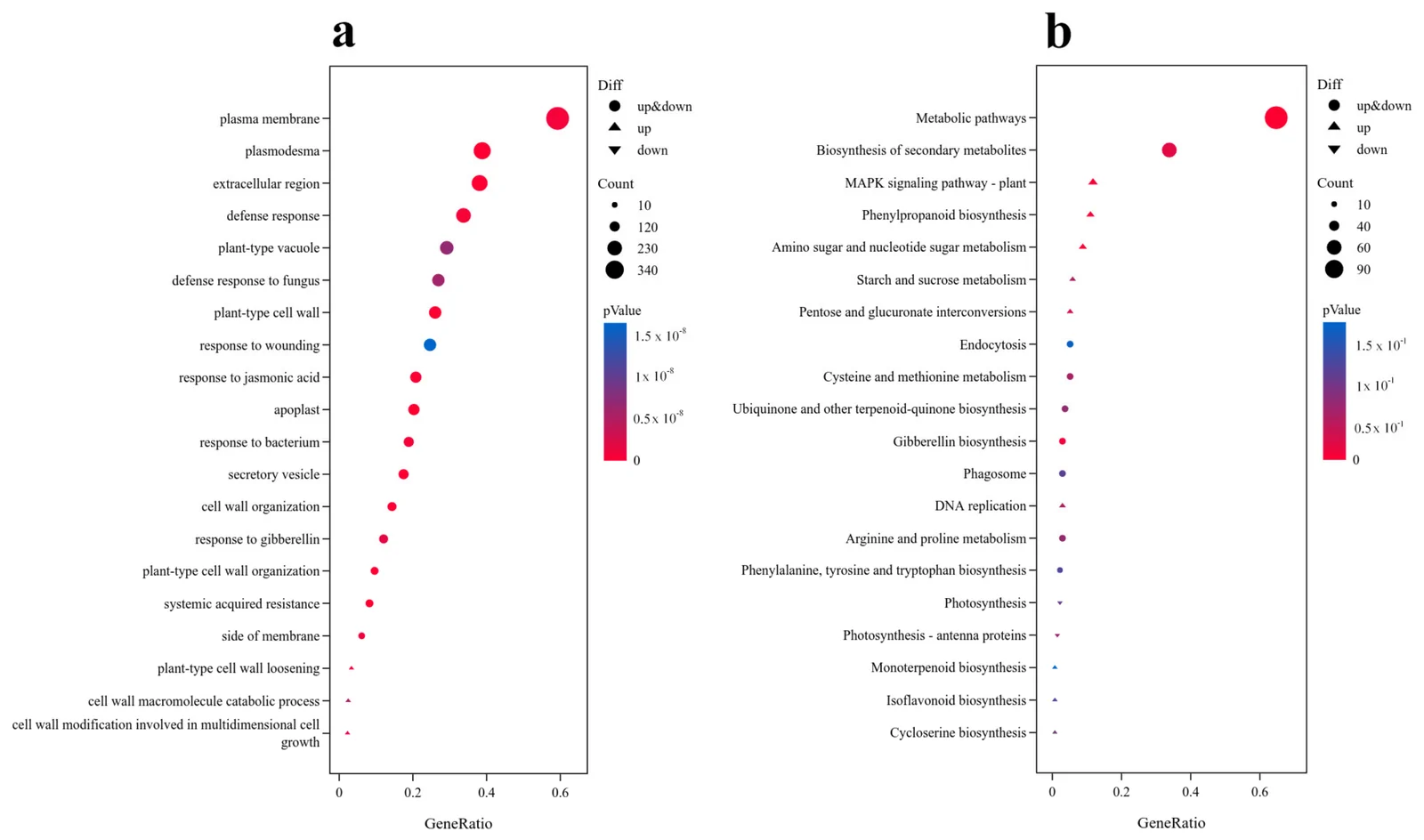
GO and KEGG enrichment analysis of the DS-1305 vs. DS-ERF23 groups. (**a**) GO enrichment bubble chart. (**b**) KEGG enrichment bubble chart. The horizontal axis represents the proportion of genes in the corresponding entry to all genes in that entry, and the vertical axis represents different gene function entries. The size of the circle represents the number of genes enriched in the corresponding entry, and the color represents the significance of enrichment.

To investigate the regulatory effect of *MnERF23* on the metabolic pathways related to drought stress in mulberry, functional annotation and pathway enrichment analysis were performed on the selected DEGs. These DEGs were enriched in the three major functional categories of biological process (BP), cellular component (CC) and molecular function (MF). When *MnERF23* was overexpressed in the absence of exposure to drought stress (CK-1305 vs. CK-ERF23), the DEGs were enriched mainly in the endoplasmic reticulum (GO:0005783), defense response (GO:0006952), and plasmodesma (GO:0009506) (Figure S8). After the *MnERF23*-overexpressing and control plants were subjected to drought stress treatment (DS-1305 vs. DS-ERF23), 339 DEGs were identified in the plasma membrane (GO:0005886), which was the most enriched functional category (Figure 5a). Some GO terms related to water scarcity and osmotic stress, such as response to water deprivation (GO:0009414), cellular response to water deprivation (GO:0042631), and response to osmotic stress (GO:0006970), were significantly up-regulated. In contrast, these GO terms were inactive in the CK-1305 vs. CK-ERF23 group, and the enrichment level of DEGs was lower. We found that many DEGs belonged to the functional categories of cell wall formation and lignin biosynthesis, such as plant type cell wall (GO:0009505), lignin biosynthetic process (GO:0009809), and phenylpropanoid metabolic process (GO:0009698). ABA is closely related to the plant drought stress response, 105 DEGs related to the regulation of abscisic acid biosynthetic process (GO:0010115) and the ABA-activated signaling pathway (GO:0009738) were identified in the functional category. There were 69 DEGs related to the response to gibberellin (GO:0009739), indicating that gibberellin may play a role in inhibiting leaf damage.

The DEGs were mapped to the KEGG database and were enriched in four categories: Cellular processes, environmental information processing, genetic information processing, and metabolism (Figure 5b). In the DS-1305 vs. DS-ERF23 comparison, the DEGs were enriched mainly in metabolic pathways (ko01100), biosynthesis of secondary metabolites (ko01110), the MAPK signaling pathway (ko04016) and phenylpropanoid biosynthesis (ko00940). These pathways were enriched in many key genes related to the plant drought stress response, and a significant number of up-regulated genes were identified, indicating that these pathways were significantly activated. Plant hormone signal transduction (ko04075) is a metabolic pathway closely related to the plant drought stress response, and compared with DS-1305, the overexpression of *MnERF23* increased the activity of this pathway in DS-ERF23. We identified 7 up-regulated genes, among which MnotChr3G00147820 and MnotChr1G00075820 are involved in the biosynthesis of ABA. Starch and sucrose metabolism (ko00500), amino sugar and nucleotide sugar metabolism (ko00520) are important metabolic pathways involved in the biosynthesis of plant cell walls. A total of 20 up-regulated genes were identified in these two metabolic pathways.

### 2.5. Overexpression of MnERF23 Activates the Expression of Drought Stress-Related Genes

ROS-scavenging enzymes play important roles in the responses of plants to drought stress. Peroxisome (ko04146) and β-alanine metabolism (ko00410) play important roles in ROS clearance, and up-regulated genes were identified in both pathways (Table S3). The cell wall is not only a passive physical barrier for plants to cope with drought stress but also a dynamic active structure that actively participates in perception, signal transduction, and adaptive regulation. Our research revealed that when the transgenic plants were not subjected to drought stress (CK-1305 vs. CK-ERF23), metabolic pathways related to the synthesis of cell wall precursors (starch, sucrose, and nucleotide sugar metabolism) were not significantly activated. However, in the DS-1305 vs. DS-ERF23 group, we identified 12 and 8 up-regulated DEGs from ko00520 (amino sugar and nucleotide sugar metabolism) and ko00500 (starch and sucrose metabolism), respectively. The overexpression of *MnERF23* activated a large number of genes in the phenylpropanoid metabolism pathway (regardless of drought), with peroxidases being the main DEGs. We detected up-regulated expression of key enzymes related to lignin biosynthesis, such as 4-coumarate CoA ligase (*Mn4CL3*, MnotChr1G00080450), hydroxycinnamoyltransferase (*MnHCT1*, MnotChr1G00043080), and O-methyltransferase (*MnCOMT2*, MnotChr1G00009070). *Mn4CL3* and *MnCOMT2* were identified only in DS-1305 vs. DS-ERF23, whose FPKM values increased by 2.86 and 5.09 times, respectively, indicating that these two genes may be induced by drought stress and *MnERF23* expression (Table S3).

In this study, qRT-PCR was used to analyze 14 DEGs that were co-upregulated with *MnERF23*. The results revealed that the gene expression dynamics were consistent with the RNA-seq results (Figure S9). The changes in expression patterns were especially consistent for key genes such as *Mn4CL3* in the phenylpropanoid metabolism pathway. These results confirm the reliability of the transcriptome data and support the prediction of the regulatory role of *MnERF23* on downstream genes.

### 2.6. MnERF23 Activates the Expression of Phenylpropanoid Metabolism-Related Genes

**Figure 6:**
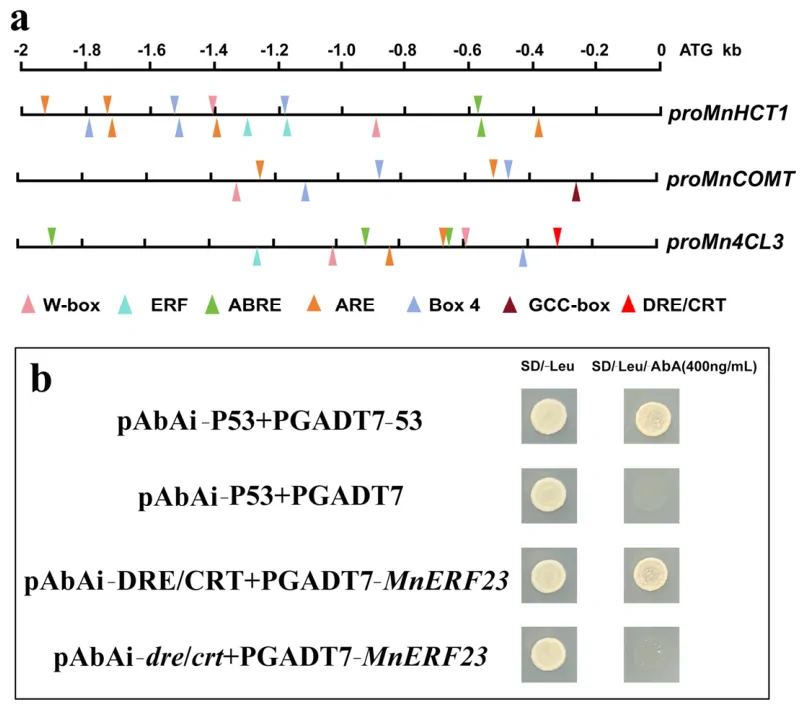
Y1H detection of binding activity between *MnERF23* and its interaction candidates. (**a**) Analysis of the *cis*-acting elements of the *Mn4CL3*, *MnCOMT2*, *MnHCT1* promoters. (**b**) Y1H verifies the binding ability of *MnERF23* with DRE/CRT *cis*-acting elements.

The overexpression of transcription factors can typically affect multiple genes or metabolic pathways. The results described above indicate that overexpression of *MnERF23* increases the expression levels of genes related to ROS clearance, cell wall synthesis, and lignin synthesis. Further analysis of the *cis*-acting elements of key enzyme-encoding genes involved in lignin biosynthesis revealed that their promoter regions retained *cis*-acting elements related to the ABA response or stress (ABRE, ARE, Box 4) (Figure 6a). We detected only DRE/CRT *cis*-acting elements (A/GCCGAC) in the *Mn4CL3* promoter region, providing the structural basis for *ERFs* to specifically recognize and regulate downstream genes.

To investigate the molecular mechanism underlying the *MnERF23*-mediated response of mulberry trees to drought stress, the promoter of *Mn4CL3* and the DRE/CRT *cis*-acting element mutation sequence were inserted into the pAbAi vector, which was subsequently cotransformed with the pGADT7-*MnERF23* recombinant vector into yeast cells (Figure 6b). The results revealed that the yeast combination of pAbAi-DRE/CRT and pGADT7-*MnERF23* could grow normally on SD/-Leu/-AbA (400 ng/mL) medium. However, the pAbAi-*dre*/*crt* and pGADT7-*MnERF23* yeast strains did not grow normally. pAbAi-P53/pGADT7-53 and pAbAi-P53/pGADT7 are positive and negative controls, respectively.

**Figure 7:**
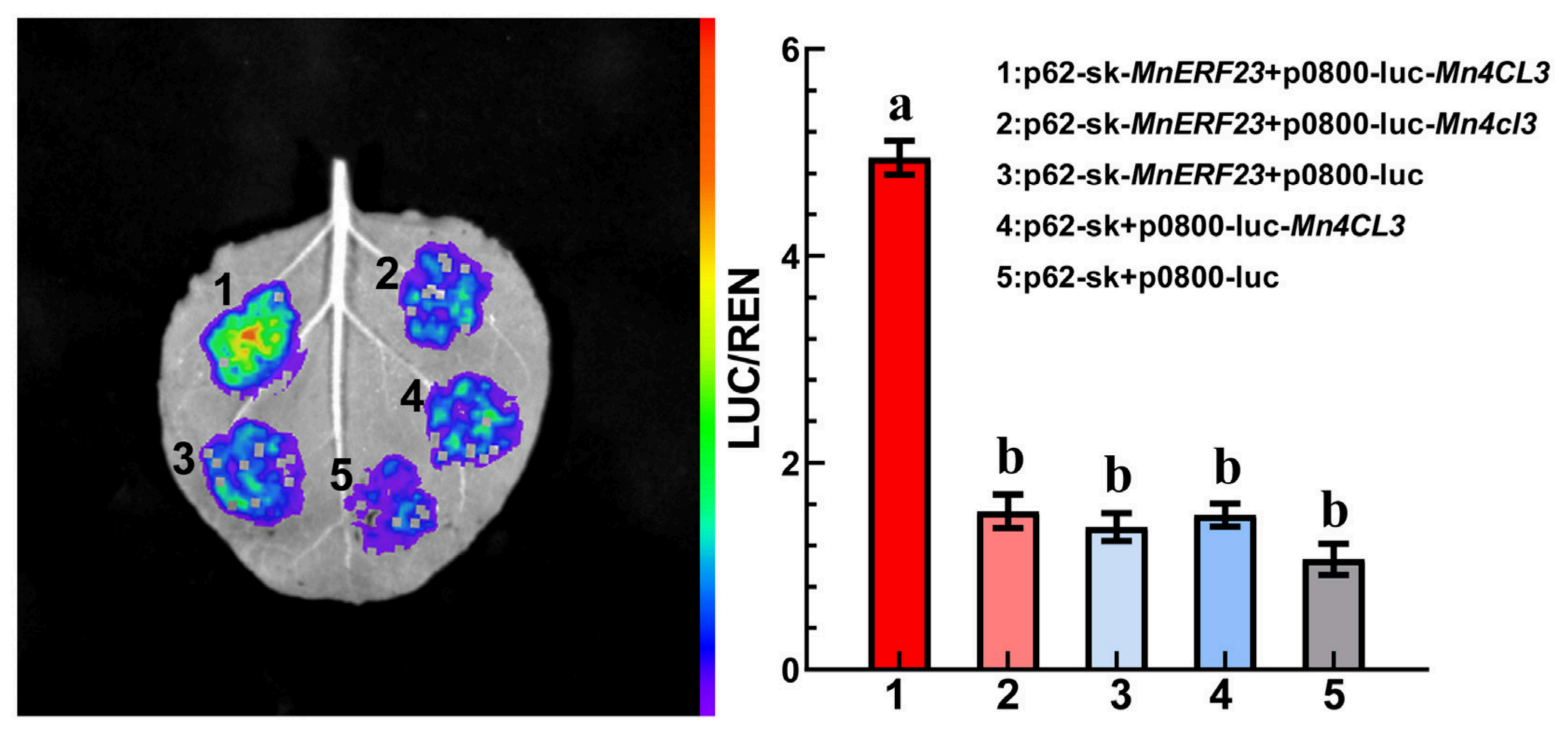
Dual-luciferase assays validate *MnERF23*-mediated activation of the *Mn4CL3* promoter. 1–5 are schematic diagrams of the effector and reporter vectors used in dual-luciferase assays. The relative luciferase activity detection results are expressed as LUC/REN ratio, different lowercase letters indicate significant differences between samples (*p* < 0.05), whereas the same letters indicate no significant differences.

We further validated the regulatory relationship between *MnERF23* and *Mn4CL3* using a dual-luciferase reporter system (Figure 7). The experimental groups consisted of p62-SK-*MnERF23* paired with either p0800-Luc-*Mn4CL3* (containing the normal DRE/CRT sequence) or p0800-Luc-*Mn4cl3* (containing a mutated DRE/CRT sequence). Three control combinations were used: p62-SK-*MnERF23*+p0800-Luc, p62-SK+p0800-Luc-*Mn4CL3*, and p62-SK+p0800-Luc. The results showed that co-transformation of the p0800-Luc-*Mn4CL3* construct with the p62-SK-*MnERF23* vector into tobacco leaves generated a strong LUC luminescence signal (Figure 7). In contrast, the fluorescence intensity of p62-SK-*MnERF23*+p0800-Luc-*Mn4cl3* was weak, indicating that mutation of the DRE/CRT *cis*-acting element prevents *MnERF23* from recognizing the *Mn4CL3* promoter.

## 3. Discussion

### 3.1. Analysis of MnERF23 Expression Pattern and Transcriptional Activation Activity

Drought stress is one of the most severe abiotic stresses worldwide and poses a dual challenge to agricultural production and global poverty reduction [22]. Drought stress can directly lead to crop deficit, inhibit photosynthesis, and cause oxidative damage, resulting in widespread reductions in the quality of grain and economic crops, posing a threat to global food security [23,24]. Drought stress in mulberry can directly lead to reduced yield, yellowing, and wilting of leaves, making it difficult to meet the requirements for direct feeding of silkworms or the preparation of industrial silkworm artificial feed [10]. Therefore, fully analyzing the molecular mechanisms underlying mulberry tree responses to drought stress is crucial for enhancing the resilience of the sericulture industry, promoting growth of income of farmers, and reducing global poverty.

Transcription factors, such as *MYB*, *NAC*, and *bZIP*, play important roles in the response of plants to drought stress and increase the ability of plants to cope with drought stress by regulating their morphological phenotypes and hormone signal transduction and activating specific protein functions [25,26,27]. The *AP2/ERF* gene family has many members, and the functions of *ERF* subfamilies in the plant drought stress response have been reported in multiple species [28,29]. For example, *HcERF5* directly regulates the ABA signaling pathway and enhances kenaf drought tolerance [30]. *MhERF113-like* is ectopically expressed in tomatoes and plays an important role in regulating plant drought tolerance by increasing the expression levels of antioxidant genes and stress-responsive genes in transgenic plants [31]. Eighty-five *ERF* genes were identified in the mulberry genome [19]. Based on evolutionary relationships, we divided these genes into 7 groups. In group I, multiple members were closely related to genes involved in plant osmotic stress regulation, such as *AtERF71*, *OsERF71*, and *PtrERF15* [16,17,32,33]. *MnERF23* is a family member that may be involved in regulating plant drought stress. The expression level of *MnERF23* increased with prolonged drought stress and peaked at 8 days. A transcriptional activation assay revealed that *MnERF23* possessed transcriptional activation activity and that the activation domain was in the C-terminal region, which is consistent with the findings for genes such as *ANT5*, *SNB* and *FZP* [34,35,36]. In the precise regulation of downstream target genes by these genes, the activation domain of the C-terminal region is indispensable, as these regions may serve as specific binding domains involved in metabolic regulation.

### 3.2. Overexpression of MnERF23 Enhances Drought Tolerance in Transgenic Plants

After the heterologous overexpression of *MnERF23* in *Arabidopsis thaliana* (OE*ERF23*-1, -2, -3), the transgenic plants were cultured on a medium supplemented with mannitol. With increasing mannitol concentration (0–200 mM), the growth of the transgenic plants improved, and the plants presented longer roots. The expression levels of genes involved in ABA signaling and the regulation of plant resistance to environmental stress, such as *AtRD22*, *AtRD29A*, *AtABI5*, *AtDREB1A*, *AtCBF4*, and *AtP5CS*, were significantly induced in transgenic *Arabidopsis thaliana* [37]. These results are consistent with the overexpression of *CqERF24* in *Arabidopsis thaliana*, which relies on the ABA signaling pathway to increase plant tolerance to drought stress, indicating that *MnERF23* may have biological functions similar to those of *CqERF24* in regulating the plant response to drought stress [38].

In this study, we transiently overexpressed *MnERF23* in the leaves of mulberry seedlings and subjected them to drought stress (the control group was transfected with the pCAMBIA1305 empty plasmid). Under stress conditions, compared with control plants, mulberry seedlings overexpressing *MnERF23* were more tolerant to drought stress, with lower levels of wilting and leaf wrinkling. Under stress conditions, plants may experience excessive accumulation of ROS, which can damage the normal activities of the plant [39]. We determined the MDA content and the activities of SOD, POD, CAT in TOE-*MnERF23* and TOE-1305 after drought stress treatment. The results were similar to the effective inhibition of H_2_O_2_ accumulation by wheat *TaERF3*, which enhanced the drought tolerance of transgenic plants [13]. Compared with TOE-1305, the overexpression of *MnERF23* significantly inhibited the accumulation of MDA and increased the activities of SOD, POD, and CAT (the activity of POD increased by approximately 2-fold).

### 3.3. Transcriptomic Analysis of MnERF23 Mediated Drought Tolerance Regulation in Mulberry

To further explore the molecular mechanism through which *MnERF23* regulates the drought stress response, a comparative transcriptomic analysis of TOE-*MnERF23* and TOE-1305 (drought-treated and normal growth groups) was conducted. Overall, the gene expression profile of the DS-1305 vs. DS-ERF23 group was more active, with a total of 554 up-regulated genes. ABA is a key hormone in the plant response to stress, and transcription factors such as *MYB*, *bZIPs*, and *WRKY* can rely on the ABA signaling pathway to increase the ability of plants to withstand abiotic stress [40,41,42]. There were more up-regulated genes in the plant hormone signal transduction (ko04075) and MAPK signaling pathway (ko04016) pathways in the DS-1305 group than in the DS-ERF23, whereas we identified only three *PP2C* genes (MnotChr1G00075820, MnotChr3G00147820, MnotChr3G00147820), and no significantly active differentially expressed genes were identified for ABA receptors and SnRK2s. We believe that owing to the effects of drought stress on the samples, some stress-related genes were activated. *MnERF23* may act on specific metabolic pathways, thus limiting the number of differentially expressed genes. In *Arabidopsis thaliana*, 1-aminocyclopropane-1-carboxylate synthase (*ACS2*) and ER receptor kinase (*ER*) play key roles in the establishment of stomatal density and accumulate in the leaf epidermis during the drought response [43,44]. We detected significant up-regulated of the homologous genes MnotChr1G00014410 (*ACS2*) and MnotChr2G00133700 (*ER*) in mulberry, indicating that these genes may be regulated by *MnERF23* and participate in the regulation of leaf stomatal density and transpiration efficiency in response to drought stress in mulberry. In addition, we identified two DEGs that may be involved in cell wall biosynthesis (MnotChr1G00077910, MnotChr1G00031520) and examined their promoter *cis*-acting element compositions (Figure S10). MnotChr1G00077910 and MnotChr1G00031520 contain elements related to drought stress response (ABRE, ARE, Box 4), and both possess ERF and MBS elements. This suggests that these two cell wall biosynthesis-related DEGs may be regulated by multiple ERF and MYB transcription factors, providing new insights for future analyses of plant drought tolerance mechanisms.

In the DS-1305 vs. DS-ERF23 comparison group, MnotChr3G00159320 and MnotChr1G00032430 are two up-regulated DEGs in the ko04146 pathway (Table S3). Analysis of promoter cis-acting elements showed that both MnotChr3G00159320 and MnotChr1G00032430 contain a large number of ABRE elements, indicating that they may be regulated by ABA and involved in ROS clearance during the plant drought stress response (Figure S10). These results indicate that MnERF23 contributes significantly to the drought tolerance of mulberry. Notably, although the function of MnERF23 has been validated, it cannot reflect long-term physiological effects due to the lack of a stable genetic transformation system. In the future, stable genetically modified plants obtained from hairy roots and other sources will be used to verify the functions of mulberry genes.

### 3.4. MnERF23 Positively Regulates Drought Tolerance in Mulberry by Activating Mn4CL3-Mediated Lignin Biosynthesis

Lignin is a type of secondary metabolic organic polymer related to the formation of plant cell walls. It can provide the necessary mechanical support for plants, ensure the smooth transportation of water and nutrients in the plant body, and increase tolerance to environmental pressures such as drought stress [45,46]. The soybean *GmeIF2B5*-*GmPRX4* regulatory axis can increase lignin deposition and enhance plant drought tolerance [47]. *PaTyDC4* promotes xylem differentiation and lignin deposition during secondary growth and confers drought tolerance [48]. The *PoWRKY31*-*PoWRKY7*5 module can induce the expression of *PoCCoAOMT*, increase lignin accumulation, improve water retention, and increase reactive oxygen species (ROS)-scavenging capacity under drought stress [49]. Overall, lignin is involved in regulating plant responses to drought stress; however, the regulatory network of lignin biosynthesis mediated by mulberry *ERF* genes is not yet clear.

Here, we investigated the lignin content of inflorescence stems in OE1305 and OE*ERF23*-1, -2, -3 (Figure S11). The results showed that compared to OE1305, overexpression plants had increased lignin content in their inflorescence stems, with OE*ERF23*-2 lignin content reaching 12.43%. This suggests that *MnERF23* may have the function of promoting lignin biosynthesis in plants. The transcriptome data also confirms this conclusion. There were 15 up-regulated DEGs in the phenylpropanoid biosynthesis (ko00940) core metabolic pathway of lignin biosynthesis, including 4-coumaric acid coenzyme A ligase (*Mn4CL3*, MnotChr1G00080450), hydroxycinnamoyl transferase (*MnHCT1*, MnotChr1G00043080), and O-methyltransferase (*MnCOMT2*, MnotChr1G00009070). *MnCOMT2* is an up-regulated gene, but based on previous research results, we believe that it may not have a biological function in the regulation of lignin biosynthesis [50]. *MnHCT1* is also an up-regulated gene, but in the *_pro_MnHCT1* sequence, we did not identify a *cis*-acting element that specifically binds to the ERF gene (Figure 6a). *4CL* catalyzes the formation of CoA thiol esters of 4-coumarate and other 4-hydroxycinnamates, which are important precursors for the biosynthesis of lignin and flavonoids [51]. Previous studies have reported that *4CL* can mediate the plant drought stress response by regulating lignin biosynthesis. For example, *Zm4CL2* and *ZmCCoAOMT* work together to regulate drought stress adaptation by modulating the expression of lignin biosynthesis-related genes [52]. Compared with the WT, the overexpression of *Fraxinus mandshurica Fm4CL-like 1* increases the lignin content of transgenic tobacco by 39.5% and enhances tolerance to osmotic stress by affecting the cell wall and stomatal development [53]. *Ta4CL91*-silenced plants became more sensitive to drought and salt stresses [54]. Chao et al. reported that mulberry MnotChr1G00080450 (*Mn4CL3*) is a clade I containing *4CLs* involved in lignin biosynthesis [50]. We found that the *Mn4CL3* promoter sequence retains a DRE/CRT *cis*-acting element (approximately 310 bp away from ATG), which is the structural basis for downstream gene regulation by *ERF* subfamily genes. Some reports have confirmed this regulatory mechanism; *JeERF1* and *TaERF1* can activate the expression of downstream osmotic stress-related genes by recognizing GCC boxes or DRE/CRT *cis*-acting elements and can participate in the response to abiotic stress [55]. *AtERF71* can also rely on DRE/CRT *cis*-acting elements to regulate downstream gene expression [56]. According to our phylogenetic tree, *AtERF71* is closely related to *MnERF23*, indicating that these genes may have similar functions and regulatory mechanisms. In this study, we aimed to elucidate the targeting relationship between *MnERF23* and *Mn4CL3*. Compared with DS-1305, *Mn4CL3* was significantly up-regulated in DS-ERF23 (with no significant change in the CK-1305 vs. CK-ERF23 groups). These findings indicate that the expression of *Mn4CL3* is induced by both drought stress and *MnERF23*. Y1H and dual-luciferase assay results reveal that *MnERF23* can rely on the DRE/CRT *cis*-acting element on the *Mn4CL3* promoter to activate its expression (Figure 6 and Figure 7). These results emphasize that *MnERF23* positively regulates drought tolerance by activating the lignin biosynthesis pathway through the regulation of *Mn4CL3*.

## 4. Materials and Methods

### 4.1. Plant Materials

The mulberry materials used in this study were sourced from the Mulberry Germplasm Resources Nursery in Anhui Province (Hefei City, Anhui Province, China). Mulberry seedlings were obtained through seed germination using the same water and fertilizer management methods. Drought stress was induced by controlling water irrigation, and sampling was initiated when the soil moisture decreased to 25% following the last watering event. The first sampling was recorded as 0 days (d), after which mulberry samples treated with drought stress for 3, 5, 8 and 10 consecutive days were collected for the detection of gene expression levels. In this study, the Columbia ecotype (Col-0) was used, and the seeds were preserved in our laboratory.

### 4.2. Phylogenetic and Promoter Cis-Acting Element Analysis

From the research of Du et al., we obtained the sequences of 85 mulberry ERF genes [19]. Sequence alignment of all ERF proteins was performed using the ClustalW tool in MEGA 7.0 software. A phylogenetic tree was constructed using MEGA 7.0 software with maximum likelihood (ML) (bootstrap = 1000). The ERF promoter intercepts a 2000 bp DNA sequence upstream of the start codon (ATG), and the online software Plantcare (https://bioinformatics.psb.ugent.be/webtools/plantcare/html/) (accessed on 8 November 2025) was used to analyze the promoter region *cis*-acting elements [57].

### 4.3. RNA-Seq Profiling

We performed RNA-seq sequencing on transient transformed mulberry leaves treated with drought for 8 d. Total RNA extraction was performed using Tiangen’s (Beijing, China) RNA extraction kit. RNA-seq was performed using an Illumina HiSeq4000 platform. The raw data has been submitted to NCBI’s SRA database with login number PRJNA1404858. Low-quality data and adapter sequences were filtered out from the reads. The filtered data were mapped onto the mulberry tree reference genome using Mnot-SWU version [58,59]. Bowtie software (v2.2.3) was used to construct the reference genome index, and clean reads were compared with the mulberry reference genome via TopHat (v2.0.12) (mismatch = 2) [60]. The FPKM values of the unigenes (fragments per kilobase of exons per million mapped reads) obtained in the previous step were calculated in Cufflinks software (version 2.2.1) [61]. RNA-seq profiling was conducted with three biological replicates.

### 4.4. Identification and Annotation of Differentially Expressed Genes

We used DESeq2 to fit the transcriptome read count data using a negative binomial distribution model and estimate gene dispersion and then performed statistical tests to identify genes whose expression differed significantly between groups [62]. The statistical results of multiple tests were all corrected using the Benjamini–Hochberg false discovery rate program (*p* value < 0.05). The differentially expressed genes obtained were subjected to Gene Ontology (GO) enrichment analysis using the topGO R package (http://www.bioconductor.org/packages/release/bioc/html/topGO.html) (version 2.60.1) (accessed on 13 September 2025). The differentially expressed genes were annotated using the Kyoto Encyclopedia of Genes and Genomes (KEGG) database (http://www.kegg.jp/kegg) (accessed on 13 September 2025).

### 4.5. Plant Transformation

The full-length coding sequence of *MnERF23* was cloned and inserted into the pCAMBIA1305-GFP vector to construct the 35S:*MnERF23* overexpression vector. The construct was subsequently transformed into *Arabidopsis thaliana* using the floral dip method [63]. The mixture was subsequently centrifuged at 4500 rpm for 15 min at room temperature to harvest the Agrobacterium strain GV3101 (pSoup) containing different constructs and cultured at 28 °C for 2 d. The bacterial cells were resuspended in infiltration buffer (10 mm MES, 10 mm MgCl_2_, 150 μm acetosyringone; pH = 5.8) and the OD_600_ was adjusted to 1.0. A syringe was used to infuse the infiltration buffer of the mixed bacterial solution into 3–4 leaves of the mulberry, resulting in transient transformation.

### 4.6. Determination of Physiological and Biochemical Indicators

Weigh 0.5 g of leaf samples from the infiltrated areas and measure the content and activity of malondialdehyde (MDA) in peroxidase (POD), superoxide dismutase (SOD), and catalase (CAT). The determination of the MDA content is often carried out using the thiobarbituric acid method. Trichloroacetic acid was added to the plant leaf samples for grinding and extraction, and the samples were centrifuged to obtain the supernatant. TBA reagent was added to the supernatant, and the mixture was heated in a boiling water bath. After the reaction, the samples were cooled and centrifuged, and the absorbance of the supernatant was measured at wavelengths of 532 nm, 600 nm, and 450 nm. The MDA content was calculated using the formula C = 6.45(A_532_ − A_600_) − 0.56 A_450_ to reflect the degree of lipid peroxidation. The guaiacol method was used to determine POD activity. The leaves were ground and extracted with precooled phosphate buffer solution and centrifuged to obtain a crude enzyme solution. To 1 mL of the test solution, 1 mL of 0.1% guaiacol, 6.9 mL of distilled water, and 1 mL of 0.18% hydrogen peroxide were added. The mixture was shaken well and allowed to react at 25 °C for 10 min, ensuring the accuracy of the reaction time. The change in the absorbance value was measured at a wavelength of 470 nm. The enzyme activity unit (U) was defined as an increase of 0.01 in absorbance per minute, and the POD activity per unit weight of the leaf samples was calculated. The activity of SOD was determined using the nitro-blue tetrazolium (NBT) method. The leaves were ground in precooled phosphate buffer and centrifuged to obtain the enzyme extract. An enzyme solution containing methionine, NBT, and riboflavin was added to the reaction system, and the mixture was allowed to react under light. By measuring the change in absorbance at a wavelength of 560 nm, the SOD activity was calculated based on the degree of enzyme activity required to inhibit NBT photoreduction by 50%. The determination of CAT activity was carried out using the ultraviolet absorption method. The leaves were ground in precooled phosphate buffer and centrifuged to obtain a crude enzyme solution. Afterward, 1.5 mL of phosphate buffer, 1 mL of distilled water, and 0.2 mL of crude enzyme solution were added to the centrifuge tube during measurement (the crude enzyme was heated and boiled as a control). The test sample was incubated at 25 °C, and 0.3 mL of hydrogen peroxide (0.1 mol/L) was added. The rate of decrease in the absorbance value of the reaction system per unit time was measured at a wavelength of 240 nm. The amount of H_2_O_2_ decomposed per unit time was calculated based on the molar extinction coefficient of hydrogen peroxide to determine the CAT activity.

Leaves were harvested before and after drought treatment, and surface contaminants were gently brushed off. Leaf discs (1 cm in diameter) were excised from the middle part of leaves (1 cm away from the main vein) and immediately weighed to record the fresh weight (Fw). The discs were then immersed in distilled water in the dark until they reached turgid weight (Tw). Afterwards, the leaf discs were oven-dried at 80 °C for 3 d to determine the dry weight (Dw). Leaf relative water content was calculated as: (Fw − Dw)/(Tw − Dw) × 100 (%) [64].

### 4.7. Quantitative RT-PCR Analysis

RNA extraction, reverse transcription, and quantitative RT-PCR (qRT-PCR) were performed as described in the product manual (Vazyme, Nanjing, China). The sequences of the primers used for qRT-PCR are listed in Supplementary Table S4. The data were normalized according to the expression levels of the *MnGAPDH* and *MnActin* housekeeping genes, and the methods were derived from the algorithms outlined [65].

### 4.8. Yeast Transcriptional Activity Assay

The division of *MnERF23* into five fragments was performed based on the conserved *AP2/ERF* domain and predicted functional regions, as previously described for ERF family proteins [66]. The full-length CDS of *MnERF23* was divided into five segments: 1-389 aa, 1-113 aa, 1-176 aa, 114-389 aa, 177-389 aa. The *MnERF23* sequence and all the fragments were inserted into the pGBKT7 vector, and the recombinant plasmid, pGBKT7 vector (negative control), and pGBKT7-VP16 (positive control) were subsequently transformed into AH109 yeast cells. The yeast cells were grown on SD/-Trp medium or SD/-Trp/-His/-Ade with X-α-gal medium for 3 d.

### 4.9. Yeast One-Hybrid (Y1H) Assay

The *Mn4CL3* promoter sequence containing the DRE/CRT element was inserted into the pAbAi vector and transformed into the Y1HGold yeast strain to construct the bait reporter strain, after which the minimum effective aureobasidin A (AbA) concentration required to suppress background growth was determined. Simultaneously, the full-length coding sequence of *MnERF23* was cloned and inserted into the pGADT7 vector. This plasmid was subsequently cotransformed with the bait reporter strain described above. Positive cotransformants were initially screened on SD/-Leu medium. To verify the specific interaction, the positive strains were further assessed on SD/-Leu medium supplemented with AbA.

### 4.10. Determination of Lignin Content in Arabidopsis Thaliana Inflorescence Stems

Place *Arabidopsis thaliana* inflorescence stems in an oven at 37 °C until completely dry. The dried plant material was thoroughly ground using a micro ball mill and then passed through an 80-mesh sieve. Approximately 0.1 g of dried, ground sample is placed in a test tube, soaked with 15 mL of distilled water, heated in a water bath at 65 °C for 30 min with occasional shaking, then filtered hot through a preweighed 0.45 μm nylon membrane. The residue is washed sequentially with deionized water, ethanol, acetone and diethyl ether (dried again). The extracted sample is digested with 25% acetyl bromide in glacial acetic acid at 70 °C for 30–60 min, which solubilizes the lignin. After digestion, the reaction is terminated by addition of sodium hydroxide, and the solution is diluted to volume. Absorbance is measured at 280 nm using a UV spectrophotometer.

### 4.11. Dual-Luciferase Imaging Assays

To validate the regulatory relationship between *MnERF23* and the *Mn4CL3* promoter, a dual-luciferase reporter assay was performed in tobacco (*Nicotiana benthamiana*) leaves. The coding sequence of *MnERF23* was cloned into the pGreenII 62-SK vector to generate the effector construct p62-SK-*MnERF23*. For reporter constructs, the native *Mn4CL3* promoter containing the DRE/CRT *cis*-acting element was inserted into the pGreenII 0800-Luc vector to produce p0800-Luc-*Mn4CL3*. A mutated version, p0800-Luc-*Mn4cl3*, was generated by introducing site-directed mutations into the DRE/CRT core sequence [67] (Table S5). Three control combinations were included: (1) p62-SK-*MnERF23*+empty p0800-Luc, (2) empty p62-SK+p0800-Luc-*Mn4CL*, (3) empty p62-SK+empty p0800-Luc. Each construct combination was introduced into *Agrobacterium tumefaciens*. Bacterial suspensions were co-infiltrated into the abaxial side of tobacco leaves using a needleless syringe. After 2–3 d of incubation under controlled conditions (dark cultivation at 28 °C), leaf discs were harvested. The Dual-Luciferase Reporter Assay Kit (Vazyme, Nanjing, China) was used to quantify the activities of LUC and REN. The final activity was expressed as the LUC/REN ratio [68].

## 5. Conclusions

In this study, we identified a mulberry *ERF* gene family member, *MnERF23*, which was induced by drought stress, and verified the biological function of *MnERF23* in regulating mulberry drought tolerance. We found that the overexpression of *MnERF23* in *Arabidopsis thaliana* could significantly increase the ability of transgenic plants to respond to drought stress and activate the expression of osmotic stress-related genes. Similar results were observed in mulberry seedlings with transient overexpression of *MnERF23*, which effectively improved the phenotype of the transgenic seedlings under drought stress. We identified 687 DEGs from the DS-1305 vs. DS-ERF23 comparison group, among which 15 DEGs were up-regulated in the phenylpropanoid metabolism pathway (including the key lignin synthesis enzyme-encoding gene *Mn4CL3*). The *Mn4CL3* promoter region contains DRE/CRT *cis*-acting elements, Y1H and dual-luciferase assay confirmed the binding activity of *MnERF23* to DRE/CRT *cis*-acting elements. Based on these findings, we clarified the regulatory mechanism through which *MnERF23*-dependent DRE/CRT *cis*-acting elements activate *Mn4CL3* expression and enhance drought tolerance in mulberry. This study contributes to the theoretical research on the regulatory network of plant abiotic stress.

## Data Availability

The data in this work have been deposited in SRA database of NCBI with accession number PRJNA1404858.

## Supplementary Materials

The following supporting information can be downloaded at: https://www.mdpi.com/article/10.3390/plants15182868/s1, Figure S1: Expression pattern of *MnERF14*, *23* under drought stress. Figure S2: Sequence alignment analysis of MnERF23. Figure S3: Expression level of *MnERF23* in transgenic *Arabidopsis thaliana*. *** significant difference at *p* < 0.001. Figure S4: The expression levels of *MnERF23* in TOE-1305 and TOE-*MnERF23*. * significant difference at *p* < 0.05. Figure S5: Measurement of water content in transient overexpression plants. Figure S6: RNA-seq mapping sequence gene structure analysis. Figure S7: Analysis of the number of differentially expressed genes between CK-1305 vs. CK-ERF23 and DS-1305 vs. DS-ERF23. Figure S8: GO enrichment analysis between the CK-1305 vs. CK-ERF23 groups. Figure S9: qRT-PCR analysis of expression patterns of genes potentially regulating the drought stress response in mulberry. Figure S10: Analysis of the *cis*-acting elements of MnotChr1G00077910, MnotChr1G00031520, MnotChr3G00159320 and MnotChr1G00032430 promoters. Figure S11: Detection of lignin content in inflorescence stems of OE1305 and OE*ERF23*-1, -2, -3. Table S1: FPKM values of 85 ERFs in mulberry under drought stress. Table S2: Overview of transcriptome sequencing data. Table S3: Screening of drought stress-related genes regulated by drought and *MnERF23*. Table S4: qRT-PCR primer sequences. Table S5: *_pro_Mn4CL3* and *_pro_Mn4cl3* sequence.
