# Supplementary material for: Mulberry ERF Transcription Factor MnERF23 Is a Positive Regulator of Plant Drought Tolerance
Source: Plants (Basel). 2026 Sep 19;15(18):2868. doi: 10.3390/plants15182868 (PMC13610394; doi:10.3390/plants15182868)
Supplement: Supplementary file 1 [file plants-15-02868-s001.zip › Table S1.pdf]

Table S1. FPKM values of 85 ERFs in mulberry under drought stress.

Gene name	Genome ID	Drought stress response related genes		
		CK	DS9	UP or DOWN
MnERF1	MnotChr1G00002120.1	0.23	0.34	UP
MnERF2	MnotChr1G00002130.1	0.29	0.026	DOWN
MnERF3	MnotChr1G00002140.1	0.09	0	*
MnERF4	MnotChr1G00002150.1	0	0	*
MnERF5	MnotChr1G00002160.1	0	0	*
MnERF6	MnotChr1G00002530.1	0.5	0.19	DOWN
MnERF7	MnotChr1G00004410.1	21.93	0.86	DOWN
MnERF8	MnotChr1G00008260.1	0.33	0.50	UP
MnERF9	MnotChr1G00008270.1	23.15	3.80	DOWN
MnERF10	MnotChr1G00009930.1	13.81	6.21	DOWN
MnERF11	MnotChr1G00010760.1	0.24	0.18	DOWN
MnERF12	MnotChr1G00018680.1	0	0.21	UP
MnERF13	MnotChr1G00018700.1	19.12	12.07	DOWN
MnERF14	MnotChr1G00021250.1	13.07	14.21	*
MnERF15	MnotChr1G00024280.1	3.19	1.27	DOWN
MnERF16	MnotChr1G00024740.1	0	0	*
MnERF17	MnotChr1G00024760.1	0	0	*
MnERF18	MnotChr1G00024770.1	0	0	*
MnERF19	MnotChr1G00024780.1	0	0.03	*
MnERF20	MnotChr1G00024800.1	0	0	*
MnERF21	MnotChr1G00034520.1	13.64	2.46	DOWN
MnERF22	MnotChr1G00040370.1	0.32	0.40	UP
MnERF23	MnotChr1G00045910.1	156.50	267.24	UP
MnERF24	MnotChr1G00048680.1	9.93	0.58	DOWN
MnERF25	MnotChr1G00050630.1	0.13	0	*
MnERF26	MnotChr1G00051560.1	0.04	0.08	UP
MnERF27	MnotChr1G00057360.1	0.17	17.65	UP
MnERF28	MnotChr1G00072700.1	0	0	*
MnERF29	MnotChr1G00073010.1	5.69	10.08	UP
MnERF30	MnotChr2G00084830.1	52.40	40.51	DOWN
MnERF31	MnotChr2G00094200.1	0.02	0	*
MnERF32	MnotChr2G00097090.1	0.73	6.19	UP
MnERF33	MnotChr2G00101690.1	1.76	4.58	UP
MnERF34	MnotChr2G00105370.1	0.04	0.42	UP
MnERF35	MnotChr2G00105380.1	0	0	*
MnERF36	MnotChr2G00105420.1	0	0	*
MnERF37	MnotChr2G00110910.1	0	0	*
MnERF38	MnotChr2G00114980.1	0.07	6.48	UP
MnERF39	MnotChr2G00118080.1	0.26	0.03	DOWN
MnERF40	MnotChr2G00120650.1	0.98	1.7	UP

Gene name	Genome ID	Drought stress response related genes		
		CK	DS9	UP or DOWN
MnERF41	MnotChr2G00123020.1	0.53	0.57	*
MnERF42	MnotChr2G00123030.1	0	0	*
MnERF43	MnotChr2G00124720.1	134.16	94.40	DOWN
MnERF44	MnotChr2G00125540.1	1.37	0	DOWN
MnERF45	MnotChr2G00126500.1	0.18	0.29	UP
MnERF46	MnotChr2G00129290.1	8.86	1.56	DOWN
MnERF47	MnotChr2G00130680.1	3.47	40.31	UP
MnERF48	MnotChr2G00135540.1	14.55	1.01	DOWN
MnERF49	MnotChr3G00141770.1	0.62	1.03	UP
MnERF50	MnotChr3G00142250.1	0.06	0.88	UP
MnERF51	MnotChr3G00150790.1	3.34	6.79	UP
MnERF52	MnotChr3G00163380.1	0	0	*
MnERF53	MnotChr3G00165080.1	24.04	226.47	UP
MnERF54	MnotChr3G00170370.1	0	0	*
MnERF55	MnotChr3G00171010.1	6.86	1.22	DOWN
MnERF56	MnotChr3G00175620.1	0	0	*
MnERF57	MnotChr3G00177510.1	0	0	*
MnERF58	MnotChr3G00177520.1	0.74	2.07	UP
MnERF59	MnotChr3G00177530.1	0	0	*
MnERF60	MnotChr3G00177540.1	0.74	2.07	UP
MnERF61	MnotChr3G00177550.1	0	0	*
MnERF62	MnotChr3G00177560.1	0	0	*
MnERF63	MnotChr3G00177580.1	0.06	0	*
MnERF64	MnotChr3G00179560.1	8.20	3.41	DOWN
MnERF65	MnotChr3G00179960.1	5.91	18.84	DOWN
MnERF66	MnotChr3G00181830.1	8.57	3.88	DOWN
MnERF67	MnotChr3G00182380.1	12.45	5.48	DOWN
MnERF68	MnotChr3G00188120.1	0	0	*
MnERF69	MnotChr4G00193700.1	8.50	11.20	UP
MnERF70	MnotChr4G00194040.1	17.59	15.04	DOWN
MnERF71	MnotChr4G00199660.1	65.42	54.43	DOWN
MnERF72	MnotChr4G00201690.1	0	0	*
MnERF73	MnotChr4G00205850.1	0	0	*
MnERF74	MnotChr4G00207000.1	0	0	*
MnERF75	MnotChr4G00211450.1	0	0	*
MnERF76	MnotChr4G00218550.1	3.05	0.05	DOWN
MnERF77	MnotChr4G00223350.1	0	0.05	*
MnERF78	MnotChr4G00223400.1	0	0	*
MnERF79	MnotChr4G00227140.1	0	0	*
MnERF80	MnotChr5G00243340.1	0	0	*

Gene name	Genome ID	Drought stress response related genes		
		CK	DS9	UP or DOWN
MnERF81	MnotChr5G00243350.1	2.31	0.53	DOWN
MnERF82	MnotChr5G00249790.1	37.21	22.66	UP
MnERF83	MnotChr6G00255790.1	4.64	4.67	*
MnERF84	MnotChr6G00269340.1	0.04	0	*
MnERF85	MnotChr6G00272280.1	18.32	11.40	DOWN
