# Supplementary material for: Mulberry ERF Transcription Factor MnERF23 Is a Positive Regulator of Plant Drought Tolerance
Source: Plants (Basel). 2026 Sep 19;15(18):2868. doi: 10.3390/plants15182868 (PMC13610394; doi:10.3390/plants15182868)
Supplement: Supplementary file 1 [file plants-15-02868-s001.zip › Table S2.pdf]

Table S2. Overview of transcriptome sequencing data.

Sample	Total Reads	Mapped Reads	Mapped Rate (%)	Unique Mapped Reads	Multiple Mapped Rate (%)	UnMapped Reads	GC content (%)	Q20 (%)	Q30 (%)
CK-1305-1	54,162,854	37,635,432	69.49	36,018,104	2.99	22,237,876	45.37	99.57	98.42
CK-1305-2	40,927,404	27,316,276	66.74	26,388,015	2.27	16,960,768	45.26	99.57	98.44
CK-1305-3	40,284,724	27,997,559	69.50	26,807,618	2.95	16,554,168	45.49	99.53	98.27
CK-ERF23-1	52,911,304	38,517,015	72.80	34,647,946	7.31	19,813,074	46.09	99.54	98.26
CK-ERF23-2	40,145,478	28,661,913	71.40	27,148,538	3.77	15,800,678	45.59	99.54	98.29
CK-ERF23-3	39,902,650	27,416,499	68.71	26,451,672	2.42	16,710,400	45.11	99.53	98.25
DS-1305-1	40,362,954	29,745,422	73.69	28,820,732	2.29	15,093,576	45.36	99.56	98.36
DS-1305-2	52,947,064	38,629,618	72.96	37,276,561	2.56	20,252,582	45.44	99.53	98.27
DS-1305-3	40,183,088	28,724,411	71.48	27,658,891	2.65	15,835,972	45.35	99.54	98.29
DS-ERF23-1	54,686,340	39,426,933	72.10	38,260,856	2.13	21,246,704	45.30	99.52	98.22
DS-ERF23-2	40,469,618	29,314,099	72.43	28,322,460	2.45	15,863,622	45.43	99.55	98.35
DS-ERF23-3	40,773,772	29,533,289	72.43	28,545,899	2.42	15,769,328	45.31	99.54	98.33
