# Supplementary material for: Mulberry ERF Transcription Factor MnERF23 Is a Positive Regulator of Plant Drought Tolerance
Source: Plants (Basel). 2026 Sep 19;15(18):2868. doi: 10.3390/plants15182868 (PMC13610394; doi:10.3390/plants15182868)
Supplement: Supplementary file 1 [file plants-15-02868-s001.zip › Table S3.pdf]

Table S3. Screening of drought stress-related genes regulated by drought and *MnERF23*.

KEGG pathway	Genome ID	DS-1305 FPKM value	DS-ERF23 FPKM value	Up or Down	log_2Fold Change	Functional annotation	p value
ko04146	MnotChr3G00159320	2.55	6.29	Up	1.23	Lipid transport and metabolism	4.87E-2
	MnotChr4G00220240	0.37	0.62	No	0.67	Lipid transport and metabolism	5.44E-1
	MnotChr1G00054160	173.39	83.61	Down	-1.15	Energy production and conversion	1.95E-4
	MnotChr1G00032430	2.20	6.74	Up	1.53	Peroxisomal	5.28E-3
ko00410	MnotChr3G00139940	23.96	14.21	No	-0.81	Energy production and conversion	3.75E-2
	MnotChr1G00006710	0.77	1.84	Up	1.17	Coenzyme transport and metabolism	4.78E-2
	MnotChr2G00093480	6.67	3.46	Down	-1.01	Energy production and conversion	2.33E-2
ko00520	MnotChr1G00029180	120.91	258.26	Up	1.01	Chitinase	1.60E-4
	MnotChr3G00156770	1.41	4.83	Up	1.69	Chitinase	6.13E-3
	MnotChr3G00156790	4.35	11.95	Up	1.38	Chitinase	2.50E-2
	MnotChr1G00077910	1.60	4.03	Up	1.24	Cell wall/membrane/envelope biogenesis	5.21E-3
	MnotChr1G00031520	20.88	46.50	Up	1.08	Carbohydrate transport and metabolism; Cell wall/membrane/envelope biogenesis	2.67E-7
	MnotChr4G00204200	0.38	3.11	Up	2.92	Alpha-1,4-glucan-protein synthase UDP-forming	5.84E-4
	MnotChr3G00174640	0.39	1.18	Up	1.50	Chitinase	1.44E-3
	MnotChr3G00156780	4.66	16.33	Up	1.73	Chitinase	2.75E-4
	MnotChr3G00174710	0.73	5.68	Up	2.84	Chitinase	1.52E-8
	MnotChr4G00197380	1.27	3.11	Up	1.22	Carbohydrate transport and metabolism	3.52E-6
	MnotChr1G00042720	58.53	159.90	Up	1.38	Chitinase	1.47E-7
	MnotChr1G00042710	90.38	242.59	Up	1.36	Chitinase	1.18E-6
ko00500	MnotChr3G00157480	1.14	12.44	Up	1.52	Periplasmic beta-glucosidase and related glycosidases	1.31E-6
	MnotChr1G00001670	2.58	11.86	Up	2.11	Endoglucanase	5.30E-5
	MnotChr2G00135830	2.04	4.61	Up	1.10	Carbohydrate transport and metabolism	2.38E-4
	MnotChr4G00211910	46.24	124.67	Up	1.35	Carbohydrate transport and metabolism	1.18E-4
	MnotChr3G00179980	2.97	9.30	Up	1.57	Endoglucanase	3.29E-5
	MnotChr3G00176140	0.95	3.00	Up	1.57	Carbohydrate transport and metabolism	8.50E-6
	MnotChr2G00124750	0.77	3.32	Up	2.01	Carbohydrate transport and metabolism	1.98E-3

	MnotChr4G00192310	1.04	3.09	Up	1.49	Glycosyl hydrolase	2.04E-3
ko00940	MnotChr1G00080450	1.94	5.56	Up	1.43	Biosynthesis of lignin	1.98E-4
	MnotChr3G00186840	28.14	96.80	Up	1.71	Peroxisomal	4.37E-7
	MnotChr3G00171830	1.07	2.56	Up	1.17	Peroxisomal	7.74E-3
	MnotChr3G00150210	21.60	67.92	Up	1.58	Peroxisomal	2.31E-7
	MnotChr2G00119350	0.94	2.27	Up	1.18	Peroxisomal	3.71E-2
	MnotChr3G00138160	4.01	10.03	Up	1.25	Peroxisomal	8.15E-5
	MnotChr4G00210500	1.11	4.18	Up	1.81	Peroxisomal	4.79E-3
	MnotChr4G00207950	0.98	2.39	Up	1.19	Biosynthesis of lignin	2.21E-2
	MnotChr4G00198140	10.92	48.66	Up	2.07	Peroxisomal	5.30E-3
	MnotChr5G00238350	0.82	2.57	Up	1.56	Energy production and conversion	2.02E-4
	MnotChr1G00043080	6.56	15.39	Up	1.16	Biosynthesis of lignin	5.40E-3
	MnotChr2G00089270	5.15	12.56	Up	1.20	Peroxisomal	2.47E-2
	MnotChr1G00009070	0.33	1.68	Up	2.18	Biosynthesis of lignin	2.15E-4
